# Where We Stand With Precision Therapeutics in Myeloma: Prosperity, Promises, and Pipedreams

**DOI:** 10.3389/fonc.2021.819127

**Published:** 2022-01-20

**Authors:** Darren Pan, Joshua Richter

**Affiliations:** Tisch Cancer Institute, Icahn School of Medicine at Mount Sinai, New York, NY, United States

**Keywords:** precision medicine, targeted therapy, multiple myeloma, novel therapies, RAS/MAPK signaling pathway, PI3K - AKT pathway, Bcl-2 inhibitor, p53

## Abstract

Multiple myeloma remains an incurable disease despite numerous novel agents being approved in the last decade. Furthermore, disease behavior and susceptibility to current treatments often vary drastically from patient to patient. To date there are no approved therapies in myeloma that are targeted to specific patient populations based on genomic or immunologic findings. Precision medicine, using biomarkers descriptive of a specific tumor’s biology and predictive of response to appropriate agents, may continue to push the field forward by expanding our treatment arsenal while refining our ability to expose patients to only those treatments likely to be efficacious. Extensive research efforts have been carried out in this endeavor including the use of agents targeting Bcl2 and the RAS/MAPK and PI3K/AKT/mTOR pathways. Thus far, clinical trials have yielded occasional successes intermixed with disappointments, reflecting significant hurdles which still remain including the complex crosstalk between oncogenic pathways and the nonlinear genetic development of myeloma, prone to cultivating sub-clones with distinctive mutations. In this review, we explore the landscape of precision therapeutics in multiple myeloma and underscore the degree to which research efforts have produced tangible clinical results.

## Introduction

Over the last two decades, the additions of proteasome inhibitors (PIs), immunomodulating agents (IMiDs), and anti-CD38 antibodies to the multiple myeloma treatment arsenal have improved survival materially (1). In recent years, further incremental gains have been made through agents with novel mechanisms, such as panobinostat, selinexor, belantamab mafodotin, and idecabtagene vicleucel. Although treatment approaches can be adjusted to patients’ comorbidities, cytogenetic risk, functional status, and response, the individual drugs that constitute current standard of care are agnostic to molecular characteristics and biomarkers. In spite of the remarkable advances, depth and duration of responses to current agents can vary widely and unpredictably due in large part to extensive intertumor and intratumor genetic variability (2). Drug-refractory relapse remains an inevitability in the vast majority of patients and each successive line of therapy produces shorter responses (3). In this context, the field has seen a renewed push for agents targeted to disease-specific characteristics.

Precision medicine approaches already have become indispensable in several other cancers, from trastuzumab in HER-2-mutated breast cancer to cetuximab in EGFR-mutated non-small cell lung cancer (4). Despite actionable mutations being identified in over three quarters of relapsed multiple myeloma patients, precision medicine has not been incorporated into standard myeloma treatment (5). The advent of genome sequencing technology has facilitated analysis of the tumor genotype with unprecedented resolution. Using these techniques, researchers have been able to compare myeloma samples to normal tissue to identify recurrently mutated pathways that may serve as additional therapeutic targets (2). Furthermore, the use of so-called “umbrella” trials, in which patients with the same cancer but different targetable biomarkers receive different treatments matched to their specific biomarker, and “basket” trials, in which patients with a variety of malignancies sharing a common biomarker are treated with a targeted treatment, has the potential to accelerate discovery of promising precision medicine candidates. The multi-armed MyDRUG protocol is one notable umbrella study in which enrolled patients are screened for targetable mutations and, if identified, are assigned to drug combinations featuring appropriate targeted agents, allowing simultaneous evaluation of multiple targeted approaches that may warrant further exploration (NCT03732703).

Precision medicine approaches hold the promise of finally incorporating this valuable genomic data to target appropriate aspects of each individual’s underlying disease biology. Actionable mutations are now regularly identified in multiple myeloma, with such abnormalities as *KRAS* mutations, t(11;14), t(4;14), and CDKN2C loss being found in approximately 29%, 20%, 20%, and 15% of cases, respectively (6–9). Here we review progress made in developing agents tailored to disease-specific biology with a focus on dissecting the degree of clinical success observed.

## Previous Clinical Pursuits: Targeted Approaches Show Mixed Clinical Results

### RAS/MAPK Pathway Inhibition

The mitogen activated protein kinase (MAPK) pathway is commonly mutated in multiple myeloma and is found in over 50% of patients (7). *KRAS*, *NRAS*, and *BRAF* mutations are generally mutually exclusive with rates of up to 29%, 24%, and 12% respectively (7, 10). MAPK pathway mutations can enhance proteasome activity, reduce cellular stress induced by PIs, and can confer resistance to PIs (11, 12). Myeloma cell lines treated with MAPK inhibitors are sensitized to the effects of PIs (12).

The V600E activating *BRAF* mutation is a poor prognostic sign, with patients harboring the mutation having a propensity for extramedullary disease (13). *BRAF*-mutated tumors such as melanoma and colorectal cancer have been successfully treated by inhibition of BRAF and the downstream MAPK kinase (MEK) (14, 15). In case reports, heavily pretreated *BRAF* V600E-mutated multiple myeloma patients experienced durable responses with either the BRAF inhibitor vemurafenib alone and vemurafenib plus the MEK inhibitor cobimetinib and served as proof-of-concept for the efficacy of *BRAF* inhibition in multiple myeloma (13, 16). Anecdotal activity was also noted with the combination of the BRAF inhibitor dabrafenib and MEK inhibitor trametinib (17, 18). The VE-BASKET study, which treated multiple *BRAF* V600-mutated tumors with vemurafenib monotherapy, observed a 33% response rate among the 9 multiple myeloma patients included (19). While these early findings show promise, whether vemurafenib efficacy can be improved by means of combination treatment or by introduction as an earlier line of therapy remains to be seen. The phase 2 CAPTUR study will treat a variety of tumor types, including multiple myeloma, according to targetable genetic abnormalities with BRAF V600-mutated disease receiving vemurafenib plus cobimetinib (NCT03297606). See **Table 1** for an overview of trials with agents that may favor particular cytogenetic or molecular profile-defined subgroups.

**Table 1:** Overview of multiple myeloma trials targeting pathways and relevant subgroups.

Targeted pathway	Drugs	Phase of study (Stage)	Treatment group - *Planned Subgroup Analysis*	NCT
RAS/MAPK	Vemurafenib, cobimetinib	Phase 2 (Ongoing)	BRAF V600 mutated malignancy	NCT03297606 (Group 12)
	Cobimetinib +/- venetoclax, +/- atezolizumab	Phase 1b/2 (Ongoing)	RRMM - *t(11;14), RAS-mutated*	NCT03312530
	Trametinib +/- GSK2141795	Phase 2 (Ongoing)	RRMM - *KRAS, NRAS, BRAF-mutated*	NCT01989598
PI3K/AKT/mTOR	Uprosertib (GSK2141795), trametinib	Phase 2 (Ongoing)	RRMM - *NRAS, KRAS, BRAF-mutated*	NCT01989598
	ONC201	Phase 1 (Completed)	Advanced solid tumors and myeloma	NCT02609230
	ONC201, dexamethasone	Phase 1 (Ongoing)	RRMM	NCT02863991
PD-1/PD-L1	Nivolumab plus ipilimumab	Phase 2 (Ongoing)	High mutational burden malignancy	NCT03297606 (Group 6)
Bcl2	Venetoclax, bortezomib, dexamethasone	Phase 3 (Completed)	RRMM - *t(11;14) and high BCL-2 expression*	NCT02755597
	Venetoclax, carfilzomib, dexamethasone	Phase 2 (Ongoing)	RRMM - *t(11;14)*	NCT02899052
	Venetoclax, daratumumab, dexamethasone	Phase 1 (Ongoing)	t(11;14)-RRMM (Part 1, 3), all RRMM (Part 2)	NCT03314181
	Venetoclax, pomalidomide, ixazomib, dexamethasone	Phase 1/2 (Ongoing)	t(11;14)-RRMM	NCT03732703 (Subprotocol E1)
	Lisaftoclax (APG-2575)	Phase 1 (Ongoing)	Relapsed/refractory heme malignancy	NCT03537482
	LOXO-338	Phase 1 (Ongoing)	Advanced heme malignancy	NCT05024045
FGFR3	Erdafitinib, pomalidomide, ixazomib, dexamethasone	Phase 1/2 (Ongoing)	t(4;14) or FGFR3 amplified RRMM	NCT03732703 (Subprotocol D1)
	Dasatinib	Phase 2 (Completed)	Relapsed or plateau-phase myeloma	NCT00429949
	AZD4547	Phase 2 (Completed)*	FGFR 1-3 mutated malignancy	NCT04439240
	EZM0414	Phase 1 (Planned)	t(4;14)-RRMM (cohort 1), t(4;14)-negative RRMM (cohort 2)	NCT05121103
CDK 4/6	Abemaciclib, pomalidomide, ixazomib, dexamethasone	Phase 1/2 (Ongoing)	CDK-activating mutation	NCT03732703 (Subprotocol A1)
IDH2	Enasidenib, pomadlidomide, ixazomib, dexamethasone	Phase 1/2 (Ongoing)	IDH2-mutated RRMM	NCT03732703 (Subprotocol B1)
MAPK, PI3K, PKC	Larotrectinib	Phase 2 (Ongoing)	NTRK1, NTRK2, NTRK3 fusion-containing malignancy	NCT02465060
Mcl1	AZD5991 +/- venetoclax	Phase 1 (Ongoing)	Rlapsed/refractory heme malignancy	NCT03218683
	S64315	Phase 1 (Ongoing)	RRMM	NCT02992483
	AMG 176	Phase 1 (Ongoing)	RRMM and AML	NCT02675452
MDM2	KRT 232, carfilzomib, lenalidomide, dexamethasone	Phase 1 (Ongoing)	RRMM - *RNA expression levels of TP53 pathway genes*	NCT03031730
	Idasanutlin, ixazomib, dexamethasone	Phase 1 (Ongoing)	del(17p) or monosomy 17-RRMM	NCT02633059

*Multiple myeloma patients eligible but none enrolled.

Cobimetinib is also being investigated independently from vemurafenib. In a preliminary report of a phase Ib/II study of relapsed/refractory multiple myeloma (RRMM), cobimetinib monotherapy demonstrated no efficacy among the 6 treated patients, but showed activity with venetoclax both with and without atezolizumab (20). The authors will be tracking the effects of t(11;14) and *RAS* mutations on response. Cobimetinib in combination with ixazomib and pomalidomide is also being further investigated in the MYDRUG umbrella protocol in NRAS, KRAS, and BRAF-mutated myeloma.

Single agent trametinib found early success in isolated individuals, including one *KRAS*-mutated patient with multiply relapsed myeloma and extramedullary disease who attained an impressive response with single agent trametinib (21). Subsequently, a retrospective analysis identified 58 trametinib-treated RRMM cases of which 51 harbored *KRAS*, *NRAS*, or *BRAF* mutations (21). Heterogeneous in both disease characteristics and treatment approach, these real-world patients with a median of 5 lines of prior therapy were treated with trametinib monotherapy, combination therapy, or in some cases monotherapy with additional agents later added. Treatment was well tolerated and produced partial responses or greater in 16 patients, with the 4 occurring on single agent trametinib further confirming its activity in this population. Still, prospective studies investigating trametinib use thus far have been sparse. Due to extensive cross-talk between the MAPK pathway and PI3K/AKT pathways, inhibition of one pathway can activate the other suggesting dual inhibition as a promising therapeutic approach (22, 23). Unfortunately, preliminary results of a phase II trial using trametinib in RRMM with addition of the AKT inhibitor uprosertib (GSK2141795) in non-responders reported an ORR of only 8% in *KRAS*, *NRAS*, or *BRAF*-mutated patients, and no responses among the 12 wild-type patients with trametinib alone (24). Addition of uprosertib increased ORR of study patients to 27%, but in the absence of further published results since this 2016 report, trial-based experience with trametinib remains limited (24).

### PI3K/AKT/mTOR Pathway

Multiple myeloma depends heavily on the bone marrow microenvironment for survival and proliferation. Interactions with stromal cells produce cytokines like IL-6, VEGF, and IGF-1 which activate the PI3K/AKT/mTOR pathway in multiple myeloma patients, initiating a signaling cascade which promotes resistance to chemotherapy and cancerous progression (25, 26). Although somatic mutations in the PI3K/AKT pathway are frequently seen in other malignancies, in multiple myeloma no activating mutations in *PI3K*/*AKT* genes have been identified (27–29). Similarly, mutations or deletions in the tumor suppressor, *PTEN*, which can disinhibit the pathway can sensitize tumors to mTOR inhibition are uncommon in multiple myeloma (30, 31). In the absence of targetable mutations, biomarkers that could predict susceptibility pathway inhibition are being actively sought and thus far trials of agents targeting the pathway have yet to adopt a precision approach.

Preclinical studies of PI3K/AKT/mTOR pathway inhibitors in multiple myeloma have long demonstrated therapeutic potential (31–35). However, initial clinical trials, many of which targeted mTOR with rapalogs such as temsirolimus and everolimus, showed muted single agent activity in multiple myeloma patients (36, 37). Everolimus plus lenalidomide, a combination which had demonstrated preclinical synergy, achieved PR or better in 21% of patients in a phase 1 study (38). Notably, a retrospective analysis found that gene expression profiles of responding myeloma patients were characterized by higher baseline expression of mTOR pathway genes (38). These findings suggest that use of microarray to identify patients with favorable gene expression profiles may represent a precision approach for future studies targeting the PI3K pathway. A phase 2 study combined temsirolimus with bortezomib in RRMM with 33% of the 43 patients responding (39). A feedback loop whereby mTOR inhibition increases IGF-1 signaling and activation of AKT may be an important mechanistic explanation for the thus far limited activity of rapalogs (40).

Rapalogs primarily inhibit mTOR’s function as a member of the multiprotein complex, TORC1. Importantly, mTOR also contributes to the function of a second complex, TORC2, whose phosphorylation substrates include AKT (41). Many newer mTOR inhibitors were rationally designed to inhibit both TORC1 and TORC2 to prevent feedback activation of AKT. Disappointingly, a phase 1 study of one such dual TORC1/TORC2 inhibitor, TAK-228, produced only one minimal response out of 26 multiple myeloma patients (42). Similarly, a phase 1 study of CC-223, another dual TORC1/TORC2 inhibitor, produced no responses out of 10 response evaluable myeloma patients, though 2 patients experienced prolonged SD (43).

Inhibition of Akt with perifosine was another approach with efficacy *in vitro* and in mouse models (34). A phase I study of perifosine, lenalidomide, and dexamethasone in RRMM produced an encouraging 73% ORR and found that responders had higher baseline bone marrow phospho-Akt levels, identifying another potential biomarker for agents targeting the PI3K/AKT pathway (44). A more recent phase III study, however, randomized patients to bortezomib and dexamethasone with or without perifosine but found no signal towards improved response rates or progression-free survival (PFS) at the first interim analysis and was discontinued (45). The pan-Akt inhibitor afuresertib has also been clinically tested in multiple myeloma, with initial monotherapy trials discontinued due to limited single agent activity (46). A basket study of afuresertib with trametinib was discontinued due to intolerable toxicities including grade 3 transaminitis and hypokalemia (47). Although the combination of afuresertib with bortezomib and dexamethasone showed a ORR of 41% in RRMM from preliminary phase 1 data, final results of the since completed study have not been published and a subsequent study of afuresertib and carfilzomib in RRMM has been discontinued (48, 49). Ongoing studies of Akt inhibitors include uprosertib with trametinib, with preliminary ORR of 27% (NCT01951495), and ONC201 as monotherapy or with dexamethasone (NCT02609230, NCT02863991).

PI3K inhibition has also been tested, although the combination of the PI3K inhibitor BYL719 and pan-PIM inhibitor LGH447 in RRMM was poorly tolerated in a phase 1 study, with 26.7% of patients experiencing grade 3 or 4 thrombocytopenia, prompting premature termination (50). With most initial clinical trials hampered by lackluster efficacy or intolerable toxicities, it remains to be seen whether the preclinical promise of the PI3K/AKT/mTOR pathway can truly be translated into tangible clinical results.

### Checkpoint Inhibition

Myeloma cells are known to overexpress PD-L1 which contributes to immune evasion (51). While preclinical evidence supported checkpoint inhibitors as a promising treatment approach, particularly when combined with IMiDs, clinical studies have yielded disappointing results (52–54). Not only have nivolumab and pembrolizumab demonstrated minimal single agent activity in myeloma, in some cases the combination of checkpoint inhibitors and IMiDs actually increased mortality (53, 55–57).

It is now clear that a better understanding of myeloma biology is required to guide checkpoint inhibitor therapy in multiple myeloma patients. In solid tumors, the issue of benefit versus immune-mediated risk with checkpoint inhibitors has been particularly favorable in tumors with high mutational burden. Tumors with high mutational burden tend to be associated with higher levels of neoantigens and tumor infiltrating lymphocytes, an environment that can be exploited by checkpoint inhibition (58). In multiple myeloma, apolipoprotein B mRNA editing enzyme (APOBEC) is a source of increased mutational burden and is associated with the MAF t(14;16) translocation (59, 60). While t(14;16) and high mutation and neoantigen burden are indicators of poor prognosis, this rationale supports the potential for these patients to derive benefit from checkpoint inhibitors (61). In the ongoing CAPTUR study, patients with high mutational burden tumors will be assigned to nivolumab plus ipilimumab (NCT03297606).

## Standout Success: The Agent Seeing Real-World Use as Targeted Therapy

### Venetoclax

Although there are currently no FDA-approved treatments for mutationally defined subsets of multiple myeloma, one agent is backed by a healthy degree of clinical trial data and has begun to see real-world use: venetoclax. Venetoclax, an oral selective inhibitor of anti-apoptotic protein BCL-2, has become a prototypical precision drug in myeloma. Currently approved in CLL and AML, venetoclax in multiple myeloma has demonstrated particular efficacy in patients harboring t(11;14) from preclinical to phase 3 trials (62). The *BCL-2* gene is highly expressed in human myeloma cell lines (HMCL) and is thought to play a role in tumorigenesis (63). However, early treatment of HMCLs noted venetoclax sensitivity primarily in the CCND1 subgroup, making the t(11;14) translocation an subgroup of interest (64). Of note, t(11;14) is the most common translocation in newly-diagnosed multiple myeloma (NDMM), seen in about 20% of cases and is associated with increased *BCL-2* expression (65, 66). In primary plasma cell leukemia, a particularly aggressive variant of multiple myeloma, t(11;14) incidence is closer to 50% and case reports support venetoclax efficacy among these patients (67–70). BCL-2 expression and dependence is further increased by dexamethasone, offering an attractive combination (71).

As with many myeloma-directed therapies with potential as targeted therapy, early trials applied venetoclax more broadly to assess for biomarker-independent activity. An initial phase 1 study of venetoclax monotherapy in RRMM demonstrated overall response rates of 20% (8). Notably, while 46% of participants harbored t(11;14), 86% of responses were found among these t(11;14) patients (8). Subsequently, the phase 3 BELLINI study randomized 291 RRMM patients to bortezomib and dexamethasone plus either venetoclax or placebo. In all-comers, the venetoclax arm improved median PFS of 22.4 months versus 11.5 months and achieved deeper responses with more patients with MRD negativity. However, patients in the venetoclax group had higher rates of grade ≥3 neutropenia (18% vs. 7% in placebo) and pneumonia (16% vs. 9%). Ultimately, the venetoclax arm experienced poorer overall survival (OS) due in large part to treatment-related deaths from infectious complications, prompting study discontinuation (72). Again, t(11;14) patients benefitted the most from venetoclax addition. Patients harboring t(11;14) or expressing high *BCL2* treated with the venetoclax combination attained deeper responses with a median PFS not yet reached compared to 9.9 months with placebo without increased mortality (72). An ongoing phase 2 study of venetoclax with carfilzomib and dexamethasone has also demonstrated safety and efficacy in RRMM, with an ORR of 75% in the 36 patients without t(11;14) and 92% in the 13 patients with t(11;14) (73). Similarly, preliminary phase 1 results in RRMM found that venetoclax, daratumumab, and dexamethasone produced a 96% ORR (all ≥ very good partial response, VGPR) in t(11;14)-only population (n = 24), while the same three agents plus bortezomib produced a 92% ORR (79% ≥ VGPR) in cytogenetically unselected patients (74). The BELLINI study along with other preliminary results have solidified venetoclax’ role as an important combination treatment option in appropriately selected patients.

Based on early efficacy data, venetoclax has begun to see off-label use in the community. One single-center study found that providers most commonly combined venetoclax with dexamethasone alone, followed by triplet regimens with PI and dexamethasone or daratumumab and dexamethasone (75). The 70 patients had a median of 3 prior lines of therapy, the majority were t(11;14) positive (86%), and the median PFS was an encouraging 13 months. Even penta-refractory patients benefitted from a PFS of 7.2 months. A different single-center study found that among 56 patients treated with venetoclax outside of clinical trials, 75% had t(11;14) and the agent was used as monotherapy or with only dexamethasone in 55% of patients while the remainder received triplets or quadruplets (76). Here, venetoclax was introduced later with a median of 6 prior lines and produced a 44% ORR but a shorter 5.8 month PFS. Notably, t(11;14) patients enjoyed longer PFS (9.7 vs. 4.2 months for t(11;14) negative, p = 0.019) and OS (not reached vs. 10.9 months, p = 0.015) (76). Another report found that between 7 Hungarian centers, 33 patients harboring t(11;14) were treated with venetoclax primarily in combination with a PI and dexamethasone either in the relapsed setting (mean 4.8 prior lines) or after suboptimal response to initial pre-transplantation induction (17 patients) (77). The authors noted that an astonishing 96% of patients responded (28% CR, 38% VGPR, 30% PR), particularly impressive when considering the refractoriness of the group. Median PFS was 15.5 months from venetoclax initiation. However, doses utilized varied widely, as only 2 patients received the 800 mg utilized in BELLINI, 1 received 600 mg, and all others received 400mg or less (77). The efficacy observed despite varied doses raises the questions of whether a reduced-intensity venetoclax regimen could represent a viable approach to improving tolerability while preserving efficacy.

A major observation from the BELLINI study was that at 800 mg daily, venetoclax was associated with significant myelosuppression and infectious, often life-threatening complications. A small recent retrospective study specifically assessed low dose venetoclax (≤250 mg/day) in combination with daratumumab, bortezomib, and dexamethasone in RRMM (78). While the 16 patient without t(11;14) had an ORR of only 31%, the 5 patients with the translocation benefited from an 80% ORR. Importantly, despite adding daratumumab to the three agents used in BELLINI, the high rates of infectious complications seen in BELLINI were not observed (no grade ≥3 pneumonia). Randomized clinical trials are needed to clarify the true benefit of reduced-dose venetoclax and whether it could represent an efficacious yet tolerable approach to precision medicine.

While the benefit of venetoclax to date has been most pronounced in t(11;14) or high *BCL-2* expressors, multiple ongoing trials continue to investigate the agent in a cytogenetically agnostic fashion. In the relapsed/refractory setting, venetoclax is being studied in combination with carfilzomib and dexamethasone and with daratumumab and dexamethasone with or without bortezomib; aforementioned preliminary data show higher efficacy in t(11;14) patients (NCT02899052, NCT03314181) (73, 74, 79). Venetoclax is also being directly compared to pomalidomide in relapsed/refractory t(11;14) positive patients (NCT03539744) and has also been included in the MyDRUG trial (NCT03732703) (80). Looking forward, a first-in-human phase 1 study of novel BCL-2 inhibitor lisaftoclax is underway in multiple myeloma and other hematologic malignancies with doses up to 1200 mg/day thus far being well-tolerated (NCT03537482) (81). LOXO-338 is another BCL-2 inhibitor being tested in a phase 1 study in advanced hematologic malignancies (NCT05024045).

## Looking to the Future: Targeted Approaches Showing Pre-Clinical Promise

### FGFR3 Inhibition

In multiple myeloma, the t(4;14)(p16.3;q32) translocation leads to deregulation of *FGFR3* and *WHSC1/MMSET* and is found in up to 20% of newly diagnosed patients (9). Patients harboring this translocation suffer from quicker relapses following chemotherapy and transplant (82, 83). The abnormality remains a poor prognostic marker even in the era of novel agents, and thus identifying effective treatments for this high-risk group remains a significant unmet need (84). FGFR3 inhibition has proved to be an effective strategy among other tumor types such as bladder cancer (85). In multiple myeloma, FGFR3 inhibition has shown preclinical promise as a form of targeted therapy, inducing apoptosis selectively in t(4;14) positive HMCLs and t(4;14) positive multiple myeloma xenografts in mice (86–89). Comparatively, FGFR3 inhibition in the clinical setting has to date been less active.

Clinically, FGFR3 has been targeted by both non-selective multikinase inhibitors and highly selective FGFR inhibitors. In multiple myeloma patients, dovitinib and nintedanib, two multikinase inhibitors which inhibit FGFR, VEGFR, and PDGFR, have demonstrated little more than a tolerable safety profile and the potential to stabilize myelomatous disease (90, 91). Dasatinib, a multikinase inhibitor effective in CML, was investigated in a phase II study of RRMM patients produced only 1 partial response out of 21 treated patients (NCT00429949).

Key to future successes of FGFR3-targeted therapies will be appropriate patient selection. Previously mentioned studies have investigated patients irrespective of mutational status or stratified by t(4;14) status. Importantly, *FGFR3* is not upregulated in all cases of t(4;14), and the translocation portends a poor outcome even in patients without altered *FGFR3* expression (92). Studies of the novel selective FGFR inhibitors erdafitinib and AZD4547 look to target patients with alterations in the FGFR3 pathway specifically, though there are currently no published results of these agents in multiple myeloma patients. Although the subprotocol of the phase 2 MATCH basket study looked to treat only FGFR3-amplified tumors with AZD4547, no multiple myeloma patients were ultimately enrolled and the subprotocol failed to meet its goal of a 16% response rate among other cancers treated (93). Erdafitinib will be added to ixazomib and pomalidomide in patients with FGFR3 activating mutations in the ongoing MyDRUG protocol (NCT03732703).

### CDK4/6 Inhibition

Cyclin-dependent kinases (CDK) 4 and 6 phosphorylate retinoblastoma protein, increasing expression of transcription factors which promote transition from G1 to S phase (94). *CDKN2C*, located on chromosome 1p, encodes a CDK inhibitor and when deleted in myeloma cells results in increased proliferation (6). Loss of *CDKN2C*, seen in 15% of myeloma cases, is associated with poorer OS (6, 95). The CDK4/6 inhibitor, abemaciclib, inhibits myeloma cell growth and exhibits cytotoxicity in a dose-dependent manner (96). Abemaciclib also induced regression of tumors in SCID models engrafted with multiple myeloma cells in part by increasing cytokines involved in NK cell recruitment and activation (97). In this model, CDK4/6 inhibition showed synergistic tumor suppression when combined with daratumumab. Patients enrolled in the MyDRUG study with 1p31 (CDKN2C) loss will be treated with abemaciclib in addition to ixazomib and pomalidomide.

Palbociclib is another CDK4/6 inhibitor which shows primarily cytostatic effects *in vitro* with multiple myeloma cell lines but enhances cytotoxicity when combined with immunomodulatory drugs, bortezomib, and corticosteroids (98–100). Thus far, the agent has only been applied in the clinical setting in a biomarker-independent fashion. A phase 1/2 study of palbociclib with bortezomib and dexamethasone in RRMM attained an ORR in 5 of 25 patients (20%) (101). Though an additional 44% achieved stable disease, the study failed to meet the ORR ≥ 28% needed to proceed to the next stage. A planned trial combining palbociclib with lenalidomide and dexamethasone terminated due to low enrollment (NCT02030483).

### Mutant IDH Inhibition

Gain-of-function mutations in isocitrate dehydrogenase (IDH) 1 and 2 results in the metabolism of isocitrate to the oncometabolite 2-hydroxyglutarate rather than α-ketoglutarate. 2-hydroxyglutarate inhibits both histone demethylase and TET2, causing an increase in both histone and DNA methylation which blocks normal cell differentiation (102). Although the use of IDH 1/2 inhibitors, ivosidenib and enasidenib, have found success in AML among the approximately 16% of IDH-mutated patients, in multiple myeloma IDH mutations are only seen in 0.5% (103, 104). Still, as IDH 1/2 mutations represent a relatively recently discovered driver mutation in myeloma, mutant IDH inhibition is now being investigated in humans, with IDH2-mutated patients in the MyDRUG protocol receiving enasidenib-based combinations (105).

### SETD2 Methyltransferase Inhibition

SETD2, a histone methyltransferase which catalyzes H3K36 trimethylation, performs a complex array of functions including DNA repair, alternative splicing, and promotion of transcriptional silencing (106). SETD2 is recurrently mutated in a number of tumor types including multiple myeloma, particularly in the relapsed and refractory setting (105, 107). In other diseases, SETD2 is thought to serve as a tumor suppressor, as mutations can hamper DNA repair mechanisms and increase mutation rates (108, 109). However, CRISPR pooled screens have found that myeloma cells are in fact dependent on SETD2 for survival (110). A small molecular inhibitor of SETD2 has demonstrated preclinical efficacy by suppressing proliferation both in myeloma cell lines and *in vivo* mouse xenografts (111). The authors also state that the agent shows *in vitro* synergy with general standard of care myeloma therapies. Clinical trials of SETD2 inhibitors are currently being planned.

### NTRK Inhibition

Fusions involving the neurotrophin receptor tyrosine kinase genes (*NTRK1, NTRK2*, and *NTRK3*) recur in cancers like gliomas, secretory breast cancer, and lung cancer (112). The oncogenic fusion protein retains the TRK kinase domain but contain part of a different gene product leading to ligand-independent constitutive activation of downstream pathways such as MAPK, PI3K, and PKC (113). The NTRK inhibitor larotrectinib has an impressive track record, demonstrating consistent and durable responses across all solid tumors harboring the fusions and has been approved for both adult and pediatric solid tumors with *NTRK* fusions (114, 115). Unfortunately, *NTRK* fusions are detected in less than 1% of multiple myeloma cases (116). Still, the MATCH basket trial is looking to enroll patients with both solid and liquid malignancies, including multiple myeloma, harboring *NTRK1*, *NTRK2*, or *NTRK3* gene fusions into the larotrectinib subprotocol (NCT02465060).

### MCL1 Inhibition

Mcl-1 is an antiapoptotic protein known to play an essential role in myeloma cell survival (117, 118). *MCL-1* overexpression confers resistance to chemotherapy, with rates of overexpression increasing at relapse (119, 120). The *MCL-1* gene is found on chromosome 1q21, along with the gene for the IL-6 receptor, and gains or amplifications of 1q21 seen in approximately 40% of multiple myeloma cases are associated with significantly shorter survival (121). Preclinical data has also demonstrated that myeloma cell lines harboring 1q21 amplifications are particularly sensitive to Mcl-1 inhibition (122). As MCL-1 upregulation is a significant mechanism of venetoclax resistance, whether co-inhibition of Mcl-1 and Bcl-2 can demonstrate clinical synergy is also of interest (66, 123).

Several Mcl-1 inhibitors are being tested in multiple myeloma in various preclinical and early clinical stages. One agent, AMG 176, induced apoptosis in hematologic malignancy cell lines and demonstrating synergy with carfilzomib and dexamethasone (124). A subsequent first-in-human phase 1 trial of AMG 176 in RRMM is ongoing and has shown preliminary tolerability (125). Common treatment-emergent adverse events were cytopenias, nausea, and vomiting. Of note, the combination of AMG 176 and venetoclax showed preclinical synergy in AML, but a dose-finding clinical trial of the combination in AML and lymphoma was suspended over safety concerns (126). AZD5991 also showed potent preclinical efficacy in multiple myeloma and AML, and a phase 1 dose-escalation study of AZD5991 with or without venetoclax is underway in relapse/refractory hematologic malignancies (127).

### MDM2 Inhibition

The enzyme mouse double minute 2 (MDM2) controls the ubiquitination of the tumor suppressor p53 and promotes its proteasomal degradation (128, 129). The expression of p53 can be decreased in a number of ways, including MDM2 overexpression, deletion of its chromosome, 17p, and inactivating p53 mutations (seen in ~8% of newly-diagnosed patients) to promote proliferation of myeloma cells (130–132). In multiple myeloma cell lines, the MDM2 inhibitor nutlin increased p53 levels and promoted apoptosis (133). Nutlin also showed preclinical synergy with bortezomib with as both increase p53 levels, but was only effective in myeloma with wild types p53 (134). As such, prolonged treatment with MDM2 inhibitors can select for a resistance line of p53 mutated cells (135, 136). In cases of p53 mutated myeloma, exclusively seen in del(17p) patients, MDM2 inhibition may still have some effect, though at higher drug concentrations (137, 138).

Deletion of 17p presents in about 10% of newly diagnosed multiple myeloma cases and within these patients approximately 37% have a p53 mutation, whereas patients without the deletion generally have wild-type p53 (138). Given the importance of wild-type p53 for the MDM2 mechanism of action, it is not surprising that the MDM2 inhibitor AMG 232, now called KRT-232, was tested in p53 wild-type multiple myeloma and solid tumors and specifically excluded del(17p) multiple myeloma (139). This phase 1 dose finding study demonstrated safety of the agent and found 240mg every 3 weeks to be the highest tolerated dose, limited primarily by cytopenias, though no responses were seen. An ongoing phase 1 study is now testing the safety of KRT-232 in combination with carfilzomib, lenalidomide, and dexamethasone in RRMM (NCT03031730).

Conversely, a phase I/II study of idasanutlin, another MDM2 inhibitor, in combination ixazomib and dexamethasone specifically in RRMM patients harboring del(17p) or monosomy 17 is also underway (NCT02633059).

## Discussion

Multiple myeloma treatment continues to evolve rapidly with numerous groundbreaking treatments. In addition to the incorporation of anti-CD38 antibodies into standard of care, CAR-T cell therapy and bispecific T-cell engaging antibodies have emerged as particularly promising treatments poised to define a new wave of myeloma-directed therapeutics. Unfortunately, as of yet, there are no signs these therapies are curative silver bullets; even patients who respond well to the newest immunotherapy approaches ultimately relapse. However, across many tumor types precision medicine approaches are often highly successful both as monotherapy and when targeted agents are combined with therapeutics with broad activity.

As our understanding of multiple myeloma becomes ever more nuanced, precision medicine allows us to provide therapy which reflects the heterogeneity we have long recognized in multiple myeloma with treatments increasingly tailored to each patient’s disease biology. We have transitioned from the original approach of classical chemotherapeutics to novel agents and now to immune-based approaches. We know that a key pathway to maximizing outcomes is to obtain deep responses through eradication of as many sub-clones as possible. As genetic drivers of myeloma are oftentimes sub-clonal, combining immune-based therapies to control bulk disease along with targeted agents to eliminate residual aberrantly driven cancer could be a conceivable pathway to cure!

The ideal precision therapies of the future should be highly efficacious in a subset of myeloma patients, have a readily measurable biomarker predictive of response, and target a mutation or other biomarker with a reasonably high incidence so as to make a significant clinical impact. Venetoclax has seen consistent success in patients with the commonly seen t(11;14) and could represent the first of conceivably many future precision medicine treatments. Agents targeting the RAS/MAPK pathway are well-positioned for clinical investigation given the frequency of *NRAS* and *KRAS* mutations in multiple myeloma and early examples of single agent and combination activity. The results of the respective RAS/MAPK-targeting arms of the MyDRUG and CAPTUR studies are eagerly awaited. Conversely, targeting biomarkers like mutant *IDH* and *NTRK* fusions, each seen in <1% of myeloma cases, are unlikely to affect the vast majority of patients even if proven useful. However, with almost 200,000 patients in the U.S. living with multiple myeloma in 2022, even low frequency regimens may provide optimal treatment for a meaningful number of patients.

At this time, many additional drugs that hold precision medicine potential are still between preclinical and first-in-human phases and the field as a whole is in its infancy. The future of precision medicine in multiple myeloma does face multiple challenges, foremost among them being myeloma’s extensive intratumoral heterogeneity. The development of this heterogeneity occurs early in myelomagenesis, even preceding clinical symptoms. In addition, this complex genetic makeup is known to change frequently over the disease course (140, 141). The success of future precision approaches will likely depend upon the use of appropriately selected combinations of both targeted and non-targeted agents, the continued use of molecular profiling to identify biomarkers, and an improved understanding of the pathways driving this disease.

## Author Contributions

DP researched and wrote the manuscript. JR edited, provided guidance regarding overall direction, and contributed to writing. All authors contributed to the article and approved the submitted version.

## Conflict of Interest

The authors declare that the research was conducted in the absence of any commercial or financial relationships that could be construed as a potential conflict of interest.

## Publisher’s Note

All claims expressed in this article are solely those of the authors and do not necessarily represent those of their affiliated organizations, or those of the publisher, the editors and the reviewers. Any product that may be evaluated in this article, or claim that may be made by its manufacturer, is not guaranteed or endorsed by the publisher.
